# Exploring the sustainability of perinatal audit in four district hospitals in the Western Cape, South Africa: a multiple case study approach

**DOI:** 10.1136/bmjgh-2022-009242

**Published:** 2022-06-23

**Authors:** Mary Kinney, Anne-Marie Bergh, Natasha Rhoda, Robert Pattinson, Asha George

**Affiliations:** 1 School of Public Health, Faculty of Community and Health Sciences, University of the Western Cape, Bellville, South Africa; 2 Maternal and Infant Health Care Strategies Research Unit, Medical Research Council of South Africa, Faculty of Health Sciences, University of Pretoria, Pretoria, South Africa; 3 Department of Neonatology, Mowbray Maternity Hospital, Cape Town, South Africa; 4 Department of Paediatrics, Faculty of Health Sciences, University of Cape Town, Cape Town, South Africa

**Keywords:** Health systems, Maternal health, Obstetrics, Qualitative study, Health policies and all other topics

## Abstract

**Introduction:**

Maternal and perinatal death surveillance and response (MPDSR) is an intervention process that uses a continuous cycle of identification, notification and review of deaths to determine avoidable causes followed by actions to improve health services and prevent future deaths. This study set out to understand how and why a perinatal audit programme, a form of MPDSR, has sustained practice in South Africa from the perspectives of those engaged in implementation.

**Methods:**

A multiple case study design was carried out in four rural subdistricts of the Western Cape with over 10 years of implementing the programme. Data were collected from October 2019 to March 2020 through non-participant observation of seven meetings and key informant interviews with 41 purposively selected health providers and managers. Thematic analysis was conducted inductively and deductively adapting the extended normalisation process theory to examine the capability, contribution, potential and capacity of the users to implement MPDSR.

**Results:**

The perinatal audit programme has sustained practice due to integration of activities into routine tasks (capability), clear value-add (contribution), individual and collective commitment (potential), and an enabling environment to implement (capacity). The complex interplay of actors, their relationships and context revealed the underlying individual-level and organisational-level factors that support sustainability, such as trust, credibility, facilitation and hierarchies. Local adaption and the broad social and structural resources were required for sustainability.

**Conclusion:**

This study applied theory to explore factors that promote sustained practice of perinatal audit from the perspectives of the users. Efforts to promote and sustain MPDSR will benefit from overall good health governance, specific skill development, embedded activities, and valuing social processes related to implementation. More research using health policy and system approaches, including use of implementation theory, will further advance our understanding on how to support sustained MPDSR practice in other settings.

**WHAT IS ALREADY KNOWN ON THIS TOPIC**

- Maternal and perinatal death surveillance and response (MPDSR) or any form of maternal and/or perinatal death review or audit is an intervention process that aims to improve health services and pre-empt future maternal and perinatal deaths; few studies have explored individual perspectives and intangible factors needed to sustain practice of MPDSR.

**WHAT THIS STUDY ADDS**

- The study examines factors that influence the sustained practice of a perinatal audit programme, a type of MPDSR, in four subdistricts in South Africa from the perspective of the users.
- The study shows that sustainability is linked to clear value-add (contribution), integration of activities into routine tasks (capability), individual and collective commitment (potential) and an enabling environment to implement (capacity), which supports contextual and local adaption.

**HOW THIS STUDY MIGHT AFFECT RESEARCH, PRACTICE AND/OR POLICY**

- Applying implementation theory through case study research enables greater understanding of MPDSR implementation.
- Implications for practice and policy include investment in good governance, innovation on how we measure successful implementation of MPDSR, improvement of skills building on data use and facilitation, integration of activities into daily practice and data systems, and conduction of more implementation research.

## Introduction

Attaining the sustainable development goal for health will require high-quality health systems that enable access and quality of care to prevent death and disease.^1^ Women and children are among the most vulnerable in societies, and their risk of death is greatest during pregnancy, childbirth and the first week after, with an estimated 4.6 million maternal and newborn deaths and stillbirths each year, mostly in low-income and middle-income countries (LMICs).^2^ Over half of these deaths (54%) could be averted by expanding coverage and quality of known interventions and innovations before, during and after pregnancy^3^; yet too often these interventions are not provided at scale or with quality in LMICs.^4^

Maternal and perinatal death surveillance and response (MPDSR) is an intervention process aimed at improving health systems for this vulnerable group (box 1). LMICs have adapted MPDSR policies and commenced their implementation,^5 6^ yet more attention to understanding and supporting implementation is needed after initial introduction to MPDSR.^6 7^ Examination of scale-up and sustainability in health, that is, continued programme activities, or implementation over a long period of time,^8^ requires consideration of different types of factors and their interlinkages.^9 10^ For example, MPDSR operates at multiple levels of the health system—national, subnational and facility—and is affected by factors at the microlevel (individual behaviour change), mesolevel (organisational culture) and macrolevel (policy and political supportive environments).^11^

Box 1 **Brief overview of MPDSR broadly and in South Africa**

MPDSR seeks to improve health systems, especially for maternal and newborn health, though a continuous cycle of identification, notification and review of maternal and perinatal deaths (surveillance), followed by actions to improve service delivery and quality of care (response).^40^ MPDSR tracks the number of maternal and perinatal deaths and identifies the main and underlying causes of death. By systematic analyses of mortality trends and the factors that contribute to each death, health system issues are identified along with solutions to prevent future deaths. The intervention process has a number of components (identify deaths, report deaths, review deaths and respond to recommendations) and involves multiple actors and teams to collect, analyse and apply the information at multiple levels of the health system.^11^ If implemented effectively, MPDSR can support the delivery of quality maternal and newborn healthcare.^71^ The World Health Organization (WHO) released global technical guidelines on maternal death surveillance and response^3^ in 2013 and perinatal audit in 2016.^4^ In 2020, WHO listed MPDSR among the essential interventions to mitigate the indirect effects of COVID-19 on maternal and perinatal outcomes.^5^ Operational guidance and tools to support MPDSR implementation were released in September 2021.^6^

In South Africa, there are separate but linked processes for MDPSR, which are outlined in the national maternity care guidelines.^72^ The National Committee for Confidential Enquiry into Maternal Deaths, established in 1998, oversees the structure of reporting maternal deaths. Every death is reported and discussed within 72 hours at the facility, and there is a confidential enquiry conducted within a month. The National Perinatal and Neonatal Morbidity and Mortality Committee, established in 2008, oversees the structure of reporting perinatal deaths. All perinatal deaths, defined as all dead babies with gestational age of 22 weeks and more (or 500 g and more), are recorded in the Perinatal Problem Identification Programme, a software and process that captures perinatal mortality and notifies deaths.^72^ Facilities are required to have regular perinatal review meetings, where deaths and data are discussed.^23 72 73^ All of these components aim to improve the quality of perinatal care and outcomes through reporting deaths and determining main causes of deaths, identifying modifiable factors, determining actions and motivating for change. Both national committees meet biannually and produce a publically available triennial report.^74 75^

To date, the literature on MPDSR mostly examines the tangible inputs required for implementation (availability of tools, focal points and committees established). While it flags the importance of the people and processes involved,^6^ few studies have explored individuals’ experiences, the dynamics of their relationships and non-tangible factors needed to sustain practice.^6^ Quality improvement interventions, including MPDSR, are complex, fluid and context-specific, requiring consideration of relationships and values among those implementing the intervention.^12–15^ Applying implementation theory may enable deeper understanding of the health policy and system factors that support the sustainability of MPDSR.^12 15–19^ Using theory, this study aimed to understand what factors promote sustained implementation of MPSDR from the perspectives of those engaged in implementation.

South Africa has been implementing perinatal audit, a form of MPDSR, since the late 1990s.^20^ Studies in South Africa assessing perinatal audit have mostly looked at the macrolevel and mesolevel and have shown the importance of team drivers or ‘champions’, institutional review, feedback and communication within the system, long history and user-friendly technology.^20–22^ Varying approaches to implementation between provinces and districts have been documented with evidence that perinatal audit can lead to health system improvements and strengthen accountability, such as clinical trainings, equipment provision and maintenance, and collaboration between primary healthcare (PHC) facilities and hospitals.^22 23^ Primary activities related to perinatal audit include the perinatal review meetings (referred to as mortality and morbidity (M&M) meetings) and the Perinatal Problem Identification Programme (PPIP) (box 1).

### Perinatal audit in South Africa

All public health hospitals conducting deliveries in the Western Cape Province in South Africa have been implementing perinatal audit for over 15 years using the PPIP.^23^ Given the long history, hospitals in the Western Cape will be a conducive environment to understanding sustained practice, considering microlevel and mesolevel factors.

## Methods

### Study design

A multiple case study design was applied to understand the ‘how’ or ‘why’ of sustained implementation.^24^ We used a multiple holistic design whereby the subdistrict was considered as a unitary whole, allowing for comparison across settings to gain insights on factors influencing sustained implementation of perinatal audit.

### Sampling

#### Sampling of subdistricts

The PPIP reporting structure in the Western Cape comprises five PPIP regions (online supplemental file 1) which are aligned to the regional hospitals with a designated regional PPIP coordinator who oversees implementation. The district level 1 hospitals manage all of the deliveries in a subdistrict, unless referral is required. Antenatal and postnatal care services take place at the PHC level. Perinatal audit considers the full continuum of care and engages both hospital and PHC staff; therefore, each case is defined as a ‘subdistrict’, with the district hospital as the host of the process. Criteria for subdistrict selection included (1) currently conducting perinatal review meetings; (2) contributing to PPIP for over 10 years; (3) a district hospital outside of Cape Town Metro, which has a unique system^23^; and (4) demonstrating at least two characteristics from a previous study on perinatal audit in South Africa: team drivers, institutional review, feedback and communication within the system.^21^ The lead author attended a provincial PPIP meeting in April 2019 with the provincial and regional PPIP coordinators to present the idea for this study, including the selection criteria and feasibility of doing this research in the different PPIP regions. Based on the criteria, stakeholder feedback at the meeting about feasibility and criteria, and interest from the regional PPIP coordinators, two PPIP regions were selected, Cape Winelands East and the Overberg (region 1) and Garden Route and Central Karoo (region 2), and then two subdistricts identified within each: cases A and B in region 1 and cases C and D in region 2. Demographics were similar across three case studies; case B had about half the population and annual births compared with the others (table 1). All subdistrict hospitals reported low levels of staff turnover.

10.1136/bmjgh-2022-009242.supp1 Supplementary data

**Table 1:** Key features of each case study

Case study	Case A	Case B	Case C	Case D
PPIP region	Region 1	Region 1	Region 2	Region 2
Population (2018/2019)	~95 000	~37 500	~95 000	~93 200
Annual births (2019)	1741	506	1360	1751
Perinatal mortality rate (per 1000 live births) (2019)	11.6	6.0	14.8	17.0
Number of PHC clinics (2018/2019)	5	5	5	5
Number of staff in subdistrict (2018/2019)	~138	~93	~205	~227
Year perinatal audit started	1999	2004	2004	2003

Data source: population, number of PHC clinics and number of staff from District Health Reports 2018/2019,^76–78^ annual births and perinatal mortality rate from PPIP database (accessed 4 March 2022), year perinatal audit started from key informant interviews.

PHC, primary healthcare; PPIP, Perinatal Problem Identification Programme.

#### Sampling of participants

Key informants were purposefully sampled based on their involvement with perinatal audit. The two regional PPIP coordinators identified key actors involved in the perinatal audit process at the district and subdistrict levels. Additional stakeholders were identified through a snowballing approach based on information provided from those interviews. For each subdistrict, we aimed to interview the medical manager, clinical manager, nursing manager, information manager or officer, manager of the maternity ward and front-line health workers who were involved in the perinatal audit process, including doctors, midwives, nurses and PHC staff. Interviews were conducted with at least 10 staff per case or until saturation had been reached, with the exception of case D, where only five staff were available. In total, 41 key informants were included (table 2 and online supplemental file 2).

**Table 2:** Demographic information about key informants

Demographic characteristics	Key informants (n=41)
Case study	
Case A	10
Case B	11
Case C	10
Case D	5
Other	5
Level of health system	
Provincial, regional, district	5
Subdistrict	16
Facility	16
PHC	4
Cadre of participants	
Provincial actors	2
Other district staff	1
Regional PPIP focal persons	2
Medical manager	3
Nursing manager	4
Clinical manager	2
Information manager	3
Quality assurance manager	1
PHC manager	1
Information officer	2
Family physician	3
Medical officer (including senior and registrar)	4
Operational manager (facility)	1
Operational manager (maternity)	3
Professional nurse	5
PHC clinic manager	2
PHC nurse practitioner	2
Sex	
Female	32
Male	9
Age group	
Below 30	2
30–49	21
Over 50	18

PHC, primary healthcare; PPIP, Perinatal Problem Identification Programme.

### Data collection

Data collection tools included a key informant interview guide and a meeting observation guide (online supplemental file 3). The interview guide focused on individual perceptions about the perinatal audit process, factors needed for implementation and team dynamics related to implementation. The meeting observation guide considered who was in attendance, information presented, and behaviours and interactions of participants.^25^ Fieldwork and data collection took place from October 2019 to March 2020, ranging from half of a day to 5 days per site. MK conducted the fieldwork and sent a summary report of preliminary findings and reflection to the research team within 1 week of visiting the site. Key informant interviews were in English and ranged from 20 min to 1 hour. All interviews were conducted individually with the exception of case D, which were done in two groups. Non-participant observations occurred at seven meetings: two provincial PPIP meetings, three subdistrict perinatal review meetings (M&M meetings), one monitoring and evaluation (M&E) meeting, and one other staff meeting.

### Data management and analysis

Interviews were recorded and transcribed. Transcripts, observation and reflection notes were compiled and analysed using Atlas.ti V.9 by MK with oversight from AG. Thematic analysis was used applying an analysis framework derived from Carl May’s extended normalisation process theory,^26^ an implementation theory used to consider broader social systems in which interventions are implemented (online supplemental file 4). Undertaking an iterative process, we developed the coding framework by analysing the data from case A using the dimensions and constructs of the extended normalisation process theory. With these findings, we identified emerging themes and gaps in the analysis framework. The codebook was revised to include descriptive factors as well as tailored to suit the intervention and related results. This revised codebook was tested and refined using the same case study as well as another (case C) before being applied to all of the data. A report was developed for each case study by MK and received inputs from all authors.

### Rigour, positionality and ethics

Measures were taken to ensure rigour of the case study approach,^24 27^ such as engagement with stakeholders prior to data collection, voluntary participation of participants, seeking peer and expert feedback, audit trail with clear mapping of the research process and triangulation of data sources. A feedback report was shared with subdistrict managers to verify results with the stakeholders. Permission to take photographs of documents and training materials was given from subdistrict health administrators, with commitment not to include sensitive information and identifiers. The lead author did not know any of the participants prior to the study but was able to develop trust with them through stakeholder engagement, including spending a few days in the subdistricts. This engagement helped to contextualise and interpret the data. Though not involved in the data collection process, other authors (A-MB, NR and RP) may have been known or familiar to some of the participants, given their involvement in the national and provincial perinatal audit processes.

### Patient and public involvement

Patients or the public were not involved in the design, conduct, reporting or dissemination plans of this study. A short report disseminating the findings was shared with subdistrict managers, district health managers, and regional and provincial stakeholders. An in-person feedback session occurred in one subdistrict with study participants.

## Results

The findings are presented according to the dimensions of the extended normalisation process theory—capability, contribution, potential and capacity (table 3).

**Table 3:** Explanatory factors enabling sustained practice of perinatal audit

Dimensions/question	Main finding	Factors identified*
**Capability**: implementation depends on its workability and integration into everyday practice. How do people integrate the work into their daily practice? Or how is it not integrated?	People have the capability to implement because activities related to perinatal audit are integrated and embedded into everyday work.	Activities are part of daily workflow. Activities are part of job expectations. Activities are part of formal training for some. Activities are linked to other meetings and QI processes. Activities are part of district support/regional outreach. Related implementation costs are embedded into existing budgets. Activities are integrated with the data system and process (eg, M&E, information unit) (C and D). Activities are part of official job descriptions (A). Activities are part of orientation (A and C).
**Contribution:** implementation depends on people’s contributions to doing the intervention by investing meaning, commitment, effort and appraisal Why do people contribute to implementation of the intervention? Or, why don’t people contribute?	People contribute to the intervention because they understand perinatal audit, value it, trust it and use it to help build and nurture relationships.	People have a common understanding of the intervention. People value it for improving service delivery, helping them learn skills, enabling them to debrief as a team. People use the review process as an opportunity to navigate professional hierarchies, hold each other accountable, improve communication and build/nurture their relationship with team members. People trust the process because the meetings are well facilitated and occur in an environment conducive to learning in a safe, non-blame environment. People also learn over time that the system works.
**Potential**: implementation depends on people’s commitment to operationalising the intervention. Why are people committed to operationalising the intervention? Or, why are people not committed?	People are passionate about their work, committed to improving the quality of service delivery and motivate each other to implement activities relating to perinatal audit.	People are passionate about their work. People are committed to providing high quality service delivery. Individual motivation stems from the desire to learn, problem solve and self-improve. Intangible incentives to attend the M&M meetings, that is, learning, debriefing, communicating. There is shared commitment to work together and improve the health system because people are invested in the area (eg, come from community or intend to continue working at the hospital for a long time). Engagement of multiple actors; when some actors are absent from the process, it makes it difficult to implement effectively. There are tangible incentives to attend the M&M meetings, that is, performance reviews (A and C) and CPD points (C and D).
**Capacity**: implementation depends on people’s capacity to co-operate and co-ordinate their actions. What gives people the capacity to implement the intervention? Or what limits people’s capacity?	People have the capacity to implement because they work in an enabling environment that supports the implementation of perinatal audits.	People work in a well-functioning hospital with sufficient and well managed material and human resources. Low staff turnover. Strong, predictable and open communication system in place between levels and staff. Good management enables a healthy organisational culture conducive to learning, innovation and accountability. Culture of data use for decision making (A, C and D). Strong social network among the staff (B).

*Factors listed means these were identified across all case studies with the exception of where indicated with A, B, C or D linked to case study assignment. Online supplemental file 7 provides a breakdown by case study.

CPD, continuous professional development; M&E, monitoring and evalutation; M&M, morbidity and mortality; QI, quality improvement.

### Capability

May posits that routine implementation depends on its workability and integration into everyday practice.^26^ Participants described perinatal audit activities as embedded into everyday workflows. In all subdistricts, the managers viewed data capturing of information about perinatal deaths as part of their routine data collection and reporting system. However, PPIP was more embedded in the information system in region 2 because the responsibility of the data capturing and analysis using the PPIP software was the responsibility of the information officer, not the clinical staff, as in region 1. The information officers reported that they would collect the PPIP data from the maternity ward at the same time as collecting data for the routine information system. Clinical staff regularly attended M&M meetings, and managers expected and monitored their participation. Participants more involved in the process, such as meeting facilitators or data capturers, reported that they had adequate time to complete the related work and considered it part of their jobs.

Perinatal audit activities were linked to ongoing processes, such as quality improvement. For example, managers tailored trainings or quality-related interventions to the identified issues during the M&Ms. Participants agreed that the ‘response’ component of perinatal audit was taken forward as part of their routine work. Discussion and action points from the M&M meetings were shared at regular management or team (ward or clinical) meetings in order to implement actions:

We'll go through the old minutes with the next Monday [bimonthly management meeting]. And they'll ask you “Did you sort that out?” and we need to give feedback on that. –Clinical manager

New staff orientations formally and informally integrated perinatal audit. Few participants reported undergoing official training on the components of the intervention, with the exception of those involved with data capture using PPIP, most of whom had received some formal training. The district health team and the regional PPIP coordinators embedded staff capacity development efforts to improve perinatal audit activities (eg, data collection) into ongoing trainings. For the most part, training was unofficial and embedded within general orientation and learning their roles on the job:

With the new operational manager of the maternity ward, we will like spot teach what M&M is and what you are supposed to record on that M&M [form] but not official training. –Maternity ward operational manager

The integration of perinatal audit into other subnational level mechanisms and activities further supported sustained practice. For example, the regional PPIP coordinators scheduled their monthly clinical outreach visit to the district hospital (to conduct routine specialist procedures) on the same day as the related M&M meeting. District teams provided materials to support implementation, such as the PPIP software and a standard operating procedure (SOP) template for how to conduct M&M meetings. In all subdistricts, the ‘ideal hospital initiative’ was being implemented, requiring a minimum number of 10 M&M meetings per year, including some perinatal-focused meetings.^28^ Costs related to implementation of perinatal audit were integrated into existing budgets. Participants did not view activities for perinatal audit as stand-alone but rather as an integral part of clinical governance.

### Contribution

Another dimension of the theory suggests that routine implementation depends on people’s contributions through investing meaning, commitment, effort and appraisal.^26^ For perinatal audit, participants had a collective understanding of the purpose but not the process and how the different components linked to each other. For example, PHC nurses did not include the surveillance system (PPIP) when asked to describe perinatal audit because they did not engage with that component.

Participants highly valued the intervention for improving service delivery, learning skills and debriefing about cases as a team. For improving service delivery, participants gave examples of change due to perinatal audit, such as additional trainings, human resource changes, the development of SOPs and acquiring additional resources. For example, one hospital permanently assigned a medical officer to the maternity ward in response to issues raised during the audit process. Another subdistrict used the audit process to advocate for additional midwives in the maternity ward. The midwives and nurses indicated that the perinatal audit process improved data capture and collection since they knew that information would be reviewed and discussed at the perinatal review meeting.

It helps us as a staff - as midwives - to be accurate with the writing of the notes because most of the time when there is an emergency, you are busy. Sometimes you forget to record … So it [perinatal death audit] helps us to improve our skills as well. –Midwife

For learning skills, participants viewed the M&M meetings as an opportunity to gain clinical skills from the referral hospital specialist (eg, obstetrician or paediatrician).

It’s almost like getting a refresher every month of at least one to three topics in obstetrics that he [outreach specialist] does. –Clinical manager

For debriefing, participants reflected that the meetings were an opportunity to collectively and openly debrief about a difficult case. Any death can be traumatic for the staff, and debriefing can help those involved understand what happened.

You need feedback on what has happened. It doesn’t help if you’ve nursed the patient and baby is gone or mom’s gone and you don’t have any feedback on what happened. –Midwife

It is [valuable] because at what other platform are we gonna discuss? One is one death too many you know. –Nursing manager

Though the team dynamics varied between subdistricts, overall participants used the review process as an opportunity to navigate professional hierarchies, hold each other accountable, improve communication, and build and nurture their relationships. An established cohesive team environment led to participants wanting to contribute to the process as part of the camaraderie felt between staff. The team approach to implementation ensured accountability and representation by multiple cadres (doctors, maternity staff, information and subdistrict management):

It’s not only a doctor driven thing. It’s a nursing and a doctor driven thing… We as the nursing staff - any category of the nursing staff - can give inputs to it [M&M]. –Nursing manager

Everybody’s got a voice there from the juniors to doctors to the sisters and I think we make everybody’s opinion count. –Clinical manager

Participants trusted the process because the review meetings were well facilitated and occurred in a safe, non-blame environment conducive to learning. The M&M meetings did not exceed the scheduled 1 hour, requiring careful preparation of cases and strategic facilitation (box 2). While only one subdistrict presented a code of conduct at the start of the meeting (online supplemental file 5), all participants believed others understood the purpose and rules of the M&M meetings. Some of the nurses and midwives still felt blamed by management and doctors during the review meetings but indicated it gets better over time. When anonymity was not maintained during M&M meetings, it was only because those involved in a case would indicate that it was their case in order to explain better what had happened, signalling they trusted the process and wanted to debrief. Of the meetings observed, the facilitator never first disclosed who was involved in the case.

Box 2 **The important role of facilitation**

Our study found that good facilitation of the perinatal review meetings was an important and common factor of sustained practice across all of the case studies and was related to multiple dimensions of the implementation theory applied. The M&M meeting facilitators enabled learning, promoted humility and inclusivity, kept time and intentionally steered the meeting to be blame-free and focused on purpose. These qualities supported sustained implementation as opposed to the alternatives.^22 34 50 55 79 80^ None of the participants reported that they underwent any specific training on management or facilitation of these meetings, with the exception of one family physician.

Effective facilitation of the review meeting can strengthen individual and collective trust in the process. It also can create an environment for learning and debriefing of an adverse outcome in a safe, non-blame environment. Although facilitation of the meetings varied between case studies, there were common factors reported and observed around what traits reflect good facilitation.

The common characteristics and qualities of the facilitators included being

- Straightforward and direct about issues.
- Approachable.
- Well respected clinician.
- Knowledgeable about the clinical protocols.
- Able to draw on personal experience.
- A teacher.
- Humble.
- Academic.

Based on observations and interviews with participants, the following recommendations may be considered to strengthen facilitation:

- Ensure careful preparation of the case before the meeting. Even though the facilitators themselves may not do the case preparation, they need to ensure that whoever is presenting the cases has done a thorough job in preparation in order to allow for a meaningful discussion. Staff involved need to have time allocated for preparation before the meeting.
- Enable local ownership in the process. In all of the case studies, a member of the clinical staff (normally doctors and/or the operational managers of the maternity ward) prepared the cases and presented the cases during the review meetings to ensure ownership.
- Remind participants about the purpose of the meeting at the start. A code of conduct or ‘audit charter’ is helpful for ensuring a blame-free meeting.^63^ In some places, this might only require an informal reminder, whereas in other places, a more formal agreement might be useful.^6^
- Steer the direction of the conversation to focus on the learning of the case. Facilitators can use the meetings as a refresher of the evidence and guidelines, emphasising clinical guidelines, importance of documentation and SOPs. By keeping the meeting focused on learning and adherence to protocols, there is less opportunity for blame.
- Demonstrate empathy. Senior staff should make a concerted effort to listen to staff who were involved in the case, prior to the meeting, and understand the reality of their experience. Facilitators who show empathy for those involved in the case and who humanise the patient by using terms, such as ‘She was a fresh stillborn’, remind the participants about the purpose of these meetings, to prevent future deaths and not to blame each other.
- Show humility. Facilitators help others learn when they can give examples of their own mistakes or experiences of an adverse outcome with what action was taken to correct it. Sharing your own experience and ability to ‘self-correct’ or advocate for change encourages others.
- Promote inclusivity. Facilitators should speak to the whole room, making eye contact with everyone rather than one individual, in order to ensure everyone feels that they are part of the team. This promotes team development and underscores the message that everyone is responsible to take forward or support others in implementing the recommendations.
- Encourage and draw on the participation of external factors, such as the clinical specialist, PPIP regional coordinator and/or subnational actors. These actors may be ‘content experts’, such as obstetricians and paediatricians, and able to bring more detailed clinical knowledge about the case. External participants can also provide an impartial perspective to the discussion.
- Keep to time. If the meeting is scheduled for an hour, guide the discussion to ensure you finish on the agreed time in order to respect everyone’s time. Going over time may prevent people from wanting to participate in the future.

By seeing how it works over time, participants knew what to expect and did not fear participation. One subdistrict experienced initial resistance to perinatal audit and found the following measures improved the process and led to sustained practice: (1) clear instructions on how to conduct meetings, (2) local adaption of the process to suit their needs, and (3) improved facilitation of the review meetings to ensure ownership and a blame-free environment by having the clinical manager lead the meeting, along with the doctor who was involved in the case.

### Potential

A third dimension of the theory posits that implementation depends on people’s commitment to operationalising the intervention. The potential for sustained practice of perinatal audit came from the individual and collective commitment by staff to deliver high-quality maternity care. At an individual level, most of the participants were very dedicated to their jobs and demonstrated pride and confidence in their clinical practice. The maternity ward staff were described as especially passionate about their work and competent:

The sisters that work in maternity they’re excellent with what they do and they’re committed. We trust that they know their job. They can manage everything. –Nursing manager

Overall commitment to high-quality service delivery was reflected by individual motivation to achieve good results and the belief that perinatal audit would help. The enthusiasm of a few committed individuals to implement the process drove others to engage and even lifted the level of commitment to perform, especially among the facilitators (box 2). In general, participants felt open to learning and new approaches.

Participants were motivated when they saw that their subdistrict statistics were among the best in the region. Subdistricts in region 2 provided continuous professional development points to doctors who attended the meetings, though this was not an incentive on its own. Financial incentives were not offered nor did people feel it was necessary (eg, per diems, tea and coffee). Collectively, there was buy-in because people saw that their engagement would yield positive change and was worth their time and their staff’s time:

You only get buy-in if people see why. If people get positive things out of it to see why am I doing this and not feel threatened and can see the learning opportunity… –Medical manager

Low staff turnover also enabled shared commitment to work together and improve the health system collectively. Many of the participants were from the subdistrict or had been there a long time, with no intention of leaving, which facilitated the motivation to improve the health system:

If your people know that this is now their hospital where they’re going to be for a few years. You try to implement things that make your life easier… But if [not], people didn’t really care about improving the system because tomorrow they’re going. –Family physician

The overall implementation process at subdistrict level was a shared task among multiple players who were committed to their role. These individuals acted as informal teams, each having a different responsibility linked to the audit process and holding each other accountable to ensuring the tasks would get done. The common characteristics of these informal teams included open and constant communication, trust in each other, and dedication to quality improvement and expectation of excellence among actors. The multidisciplinary nature of the process demonstrated shared commitment among all actors engaged. Some key actors were consistently absent from the observed processes, notably emergency medical services (EMS) and district health management. Three of the subdistricts reported that an EMS representative would normally attend the perinatal meeting or would attend if asked, but even in these settings, some participants expressed frustration when they did not attend, given their important role in the referral process. Direct engagement from the district health management team was limited in the perinatal audit process. Subdistricts in region 2 reported that the district comprehensive health manager would sometimes attend; subdistricts in region 1 reported no engagement from the district office. These subdistrict managers indicated that information related to perinatal audit would be reported to them in other meetings as relevant.

### Capacity

The final dimension of the theory considers that implementation depends on people’s capacity to co-operate and co-ordinate their actions. Across the case studies, participants described working in an environment that supports the implementation of perinatal audit. These subdistricts have well-functioning hospitals with highly competent staff, at the management and clinical levels. Resources were already in place to implement perinatal audit, that is, staff capacity, data capturing forms, computers and available space (meeting room). Some participants reported budget constraints to implement actions identified through the audit process, that is, human resources and equipment procurement. For example, all subdistricts, except for case B, reported not having enough staff in the maternity wards. Subdistrict managers responsible for addressing these challenges considered these challenges as part of the broader budget management and constraints, as demonstrated by this quote:

Most of the time what comes up in these perinatal reviews is the number of staff. But I must look at the budget… a professional nurse in maternity ward is expensive… My hands are tied because this is like my budget. How am I going to cut it? –Nursing Manager

The subdistricts demonstrated professional work environments with clear and regular communication. Communication channels between the district hospital and the regional referral hospital included a range of mediums, for example, phone, email and WhatsApp. Participants felt there was open communication between team members and health system levels, which made it easier to share information. For example, participants indicated they could call the regional hospital and speak directly to a specialist (eg, obstetrician or neonatologist) and get guidance over the phone about how to manage a case. This type of open communication strengthened trust and joint responsibility between health system levels and contributed to a healthy organisational culture conducive for implementation of perinatal audits.

All case studies demonstrated strong data use for decision making more generally with maternity-related statistics visible in the hospital and regular M&E meetings at subdistrict and district levels to inform health system planning. PPIP data use for decision making varied between the regions. Region 1 did not use the PPIP data for local decision making, whereas region 2 had a strong system of using the data and information from PPIP, as demonstrated by this quote:

The M&E - where we have all the role players together - it’s great because using the [PPIP] data then we can say “this is the issue with the transport from [city]. This is the issue with the CPAP (continuous positive airway pressure) at [case D] hospital” and immediately you can address more things. –Regional PPIP coordinator

The overall management was excellent, as observed and reported. The top-level managers (medical manager, clinical manager and matron) demonstrated strong managerial skills, which fed into good management of others on their team (other managers and operational ward managers). The managers interviewed were supportive and protective of their staff. Managers reported one-on-one meetings with those involved in a perinatal death prior to M&M meetings in order to demonstrate support and identify issues before the group meeting. By working alongside their staff, managers were visible and able to mentor staff, including in perinatal audit-related activities, such as how to correctly complete the PPIP forms and apply learning from the M&M meetings. The regional PPIP coordinators also had strong management skills and served as mentors to the staff in subdistricts, with an aim to grow champions to strengthen implementation.

I think it’s just lead by example, be open, be a good example why, and just care. Care for your patients, care for your staff. –PHC operational manager

It’s like a tree. So you start with the stem and a couple of branches and you’re adding leaves all the time. So - like the other day when I went to [district hospital] when [doctor] presented the PPIP data himself. It’s not that the tree is suddenly full of leaves, but it’s a slow process of adding people and getting them enthusiastic. –Regional PPIP coordinator

## Discussion

This study presents factors that promote sustained practice of perinatal audits from the perspectives of the users in four subdistricts in the Western Cape, South Africa. Using the normalisation process theory, we learn that implementation is supported by integration of activities into routine tasks (capability), clear value-add (contribution), individual and collective commitment (potential), and an enabling environment to implement (capacity). To place these results in relation to the literature, we will apply a conceptual implementation framework developed specifically for MPDSR.^6^ The framework includes three cross-cutting health systems lenses: service delivery (tangible inputs), societal (social relationships) and systems (interactions over time and levels).^6 10 11^ Box 3 presents implications and recommendations.

Box 3 **Policy and programme implications and recommendations**

1. Invest in overall good governance. This study shows the importance of an overall enabling environment with good leadership, strong management, open communication and data-driven decision making. Perinatal audit has the potential to strengthen individual staff capacity and motivation and even helps to build team relationships. Linking perinatal audit to other accountability mechanisms, such as key performance areas and staff performance assessments, can ensure individuals participate and actions are taken forward. Improving overall health system governance can strengthen MPDSR implementation, just as a functional MPDSR programme can strengthen the health system.
2. Innovate how we measure successful implementation. The benefits of the perinatal audit programme, as perceived by users, go beyond tangible changes to include social and individual processes, such as health worker motivation, mutual accountability, confidence in clinical skills and cohesive team building. These factors are also needed for health systems and can determine the success or failure of quality improvement interventions more generally.^50 61 81 82^ So often in the literature and global guidelines, the impact of MPDSR is only measured by considering output and outcome indicators, with little evidence of impact.^30 40 47 73^ Redefining implementation success to consider the perceived values of MPDSR programmes, for example, navigating hierarchies, learning and debriefing after an adverse case, may promote sustained practice.
3. Improve skills building on data use and facilitation. Specific skills are needed to implement MDPSR programmes, including preparation and facilitation of perinatal review meetings and data collection and analysis. In this study, most people did not report undergoing specific training on how to facilitate or engage in the perinatal review meetings. Also, there had not been a PPIP training in over 5 years. Materials already exist to support these skills development through the Perinatal Education Programme in South Africa.^36 37 72^ Targeted preservice and in-service training and mentorship programmes should incorporate these skills development.
4. Integrate activities related to MPDSR into daily practice and data systems. Our study shows that people had the capability to implement activities related to perinatal audit because it was part of the work they were already doing and were expected to do. Embedding tasks related to MPDSR in job descriptions, orientations and ongoing activities can support sustainability. Additionally, integrating PPIP data use at subnational levels through M&E processes and M&M meetings promotes sustainability. PPIP, as a tool (forms, software and outputs), was more valued and more embedded in region 2, where information officers analysed and presented the PPIP data at subdistrict and district M&E meetings.
5. Implementation research. More research using health policy and systems research approaches will be needed to explore the implementation process in different contexts, over time, and the impact of the COVID-19 pandemic. Since most studies on MPDSR implementation focus on tangible factors,^6^ there is a need to expand our knowledge of implementation considering theory-based approaches, allowing further understanding of the complex interplay and change dynamics linked to the success and sustainability of the intervention.

### Service delivery lens: inputs needed for implementation

Our study validates the need for tangible system inputs, such as focal points and regular meetings,^5 6 29–33^ and shows that for sustainability, integrating these into routine practice and systems gives people the capability to sustain implementation. Organisational incentives (refreshments, per diems and continuous professional development points) did not appear to contribute to sustained participation in our study, though incentives have been identified in other settings.^6 34^

Training and supervision can also promote sustainability.^6 7 9 22 35^ This study shows that training was mostly informal and integrated, especially after initial introduction. The long history and scale of the Perinatal Education Programme in South Africa, which includes perinatal audit as part of the curriculum, may also have contributed to sustained practice,^36 37^ though it was not specifically identified or explored in this study. Low staff turnover and continuous supervision by the regional PPIP coordinators helped maintain skills and knowledge. As with other studies, participants believed they had the skills needed to fulfil their responsibilities related to perinatal audit.^38 39^ Nonetheless, few people had a full a grasp on all of the steps in the audit cycle and how they linked. A clear explanation of the components of MPDSR and a list of competencies required for implementation remains elusive in the global literature.^6 40^ The lack of a common understanding of MPDSR implementation, as reflected in global literature and the users of the intervention in this study,^6^ may impede our ability to demonstrate effectiveness and sustainability of the intervention process, which is not a problem unique to MPDSR.^41^

While no standard minimum requirements of human and material resources for MPDSR implementation have been identified,^6^ sufficient and well-managed human and material resources may contribute to sustainability.^9 34 42 43^ In our study, the belief that there were sufficient resources to respond to identified actions may have reflected the Western Cape Province’s rich and unique experience of health-system transformation and a relatively well-functioning overall health system.^44^ Future research may want to analyse budgets and expenditures relating to perinatal audit to validate these beliefs.

### Societal lens: interactions between those involved

External influences can affect the perceived legitimacy of MPDSR.^6^ Our study finds that the expectation of reporting and engagement from the regional PPIP coordinators, along with other accountability mechanisms, that is, the ‘ideal hospital’, gave legitimacy to participate in perinatal audit, as found in other South African studies.^21–23^ The clear and intentional linkages to the routine information system in region 2 added another layer of accountability, as shown in studies from India^45^ and Malawi.^46^ The integration of perinatal audit into other processes embedded activities into the broader frame of clinical governance rather than as a stand-alone activity, further supporting the presumption that MPSDR should be implemented along with other clinical governance practices.^6 7 47^

The belief that the intervention achieves its desired outcome, also called ‘value proposition’, promotes sustainability.^20 48^ While many studies have shown positive outcomes from MPDSR,^6^ few have also linked this to buy-in and sustainability.^6 20 49–52^ Our study shows that seeing the benefits of engaging over time enabled people to buy in and become more committed to perinatal audit, ultimately improving implementation.^6^

Individual motivation to implement MPDSR is critical for sustained practice.^8 26^ Our study confirms what others have found regarding intrinsic motivation related to MPDSR,^6^ such as passion for maternity care and commitment to improving the quality of service delivery. Additionally, we learnt that users valued the opportunity to debrief as a team after difficult cases in a safe and trusted space. As for extrinsic motivation, people appreciated the opportunity to learn clinical skills through MPDSR.^6^ Individual motivation, buy-in to MPDSR and general commitment to their jobs and quality improvement are linked.^21 51 53^ Our study finds that clear tools, supportive supervision and continuous oversight from subnational actors improved individual confidence in implementation, and this aligns with the quality improvement literature.^35^

Sustainability is supported when people have a common understanding about an intervention; they value it, trust it, and use it to help build and nurture relationships.^8 54^ The nature and quality of teams, including the hierarchies, mentorship, teamwork, facilitation and management, all played a role in MPDSR implementation.^6^ Multidisciplinary team engagement is widely acknowledged as an enabler,^6 22 55^ but less studied are the small informal teams that are core to implementation.^6^ Our study confirms the importance of such teams for sustainability, especially when they operate in an environment with clear communication channels and mutual respect.^21 56^ The importance and value of investing and strengthening these informal teams require more attention for those seeking to strengthen MPDSR implementation.^40^

### Systems lens: things that trigger change

Local adaption of an intervention is a core element of sustainability.^39 57–59^ The contextual and local adaptation of the MPDSR process has been well documented in South Africa and other LMIC settings,^6 21 60^ is promoted by the WHO,^40^ and aligns with broader quality improvement approaches.^35 61^ Our study shows variability in implementation processes between sites, and that subdistricts continuously tailored the process to the capacity, interests, and needs of the actors involved.

Implementation culture profoundly influences MPDSR and its sustainability,^6 22 55 62 63^ and multiple frameworks seek to support how to overcome the blame culture specifically.^51 63^ Our study validates elements that prevent blame in MPDSR, such as strong leadership, codes of conduct, participation, openness, professionalism and self-reflection.^7 21 38 51 60 64 65^ Strong leaders or ‘champions’ are a critical factor in MPDSR sustainability,^6 20 21^ and our study goes further to identify traits and motivations of these individuals (Box 2), which align with common aspects found in good leaders or managers.^66 67^ Strong, predictable and open communication systems, along with effective management, enable a work culture conducive to learning, innovation and accountability linked to perinatal aduit.^8 39 61^ The observed positive implementation culture of perinatal audit in this study took time to nurture and also was part of a wider effort to strengthen quality improvement, self-reflection and joint responsibility.^23 44 68^ The Western Cape Department of Health’s governance approach of collaboration, integration and multisectoral engagement may have influenced the implementation of the perinatal audit programme and enabled it to benefit from and contribute to the broader health system.^44 68 69^

By focusing on explanatory factors, our study provides a deeper understanding into how and why MPDSR routinely works in the Western Cape. While our study confirms findings from other studies in South Africa,^21–23 65^ the focus at the mesolevel and microlevel allowed for more understanding on how individuals and teams perceive perinatal audit implementation.

### Application of the extended normalisation process theory

The adapted version of the extended normalisation process theory provided a structure to understand and explain an environment with sustained implementation.^26^ May argues that his theory is useful for understanding complex implementation processes in the ‘real-world’ environment where they are implemented. The MPDSR process is complex, with many parts or steps, different actors at multiple levels, and the interaction between people, teams and the health system.^11^ By applying a health policy and systems approach, we were able to unpack the contextual factors and underlying mechanisms that might render MPDSR to be sustained.^15 35 70^ Using theory, we explored issues such as trust, credibility and hierarchies shaped by the power relations between stakeholders even when the implementation process slightly varied between cases.

For the maternal and child health community, this study demonstrates the value of using theory as a means to understand complex implementation.^12 15–19^ Most studies in maternal and child health fall under the service delivery lens, measuring the tangible markers of an intervention.^10^ Our study confirms that factors enabling sustained practice of MPDSR require investments in the societal and systems lenses, or intangible elements of the health system, and this will require qualitative research approaches.^6^

### Limitations

This study collected information on perinatal mortality audit, which is a sensitive topic, given the nature of exploring adverse incidents by reporting data on deaths as well as reviewing the situation surrounding the death. Participants may not have shared their actual understanding of the process or experience or may have changed their behaviour during the observed review meetings. Through individual interviews, this study included the perspectives of front-line health workers, subdistrict health management and regional actors involved in the PPIP process. District management staff were not available for interviews (scheduling conflicts), and not all of clinical staff and subdistrict managers were included. Data collection stopped at the end of March 2020 due to the COVID-19 pandemic and related restrictions. This unfortunately prevented further data collection, including observation of additional meetings, and timely validation meetings with the subdistricts.

To ensure rigour and trustworthiness, triangulation of the different data sources was used to verify and validate information including field notes, observations and follow-up interviews with specific people. There was possible interpretive bias of the lead researcher (MK) due to issues of reflexivity and specific interests. However, the interviews were conducted using a semistructured interview guide and data were analysed using an implementation theory and adapted analysis coding framework with review from all authors.

## Conclusions

The sustainability of MPDSR relies on societal and health systems elements as well as tangible markers of implementation and their interactions. Through case study research in four subdistricts of the Western Cape, South Africa, this study reveals the importance of contextual and local adaptation. To sustain perinatal audit, related activities were embedded into everyday work (capability), and the users valued and understood the process (contribution). Elements relating to context also played an important role, including the skills and motivations of the individuals involved (potential) as well as an enabling environment with adequate resources, data use, management and communication (capacity). This study applies an adapted implementation theory to understand sustainability highlighting the complex interplay of actors, their relationships and context. More health policy and system research will advance our understanding on how to support sustained practice of quality improvement interventions.

## Data Availability

Data are available upon reasonable request. The data (case study reports and transcripts) are available from the corresponding author on request.
