# Supplementary material for: Exploring the sustainability of perinatal audit in four district hospitals in the Western Cape, South Africa: a multiple case study approach
Source: BMJ Glob Health. 2022 Jun 23;7(6):e009242. doi: 10.1136/bmjgh-2022-009242 (PMC9226866; doi:10.1136/bmjgh-2022-009242)
Supplement: Supplementary data [file bmjgh-2022-009242supp001.pdf]

Supplementary files
Title: Exploring the sustainability of perinatal audit in four district hospitals in the Western Cape, South Africa: a multiple case study approach.

Supplementary files:

- Supplementary file 1: Description of settings
- Supplementary file 2: Distribution of participants and meeting observations
- Supplementary file 3: Data collection tools
- Supplementary file 4: Analysis framework and constructs
- Supplementary file 5: Code of conduct example
- Supplementary file 6: Example of a well-facilitated perinatal death review meeting
- Supplementary file 7: Mapping of specific factors by case study

Supplementary file 1: Description of settings by case study

Table S1.1: Setting of case studies

**Sub-district**	**Case A**	**Case B**	**Case C**	**Case D**
PPIP region	Region 1	Region 1	Region 2	Region 2
Population (2018/19)	~95,000	~37,500	~95,000	~93,200
Annual births (2019)	1741	506	1360	1751
Perinatal mortality rate (per 1000 live births) (2019)	11.6	6,0	14,8	17,0
Sub-district facility structure	Facilities: District Hospital, five clinics	Facilities: District Hospital, five clinics	Facilities: District Hospital, three clinics	Facilities: District Hospital, five clinics
Human resources	~138 total staff in sub-district<br>18 nurses in maternity ward, including professional nurses (who are permanent staff)<br>14 doctors (work on rotation in maternity ward)	~93 total staff in sub-district<br>3 nurses in maternity ward during a shift one advanced midwife who is permanent staff)	~205 total staff in sub-district<br>18 nurses in maternity ward, including professional nurses (who are permanent staff);	~227 total staff in sub-district<br>5 nurses in maternity ward during a shift (two advanced midwives)

Sub-district	Case A	Case B	Case C	Case D
	Low turnover in staff	4 doctors (work on rotation in maternity ward)<br>Low turnover in staff	9 doctors on rotation; one doctor maternity ward permanently for past 6 months)<br>Low turnover in staff	4 doctors (work on rotation in maternity ward)<br>Low turnover in staff
History of PDA implementation	Long history (1999)<br>Regional PPIP focal point was among first PPIP users starting in 1999<br>No significant changes in implementation process	Long history (before 2004)<br>Regional PPIP focal point was among first PPIP users starting in 1999<br>No significant changes in implementation process	Long history (before 2004)<br>Some changes to process (who completed forms, structure of M&M meetings, using PPIP data at sub-district M&E meetings in 2015	Long history (2002/3)<br>Many changes to the process overtime (PPIP software upgrades, structure of M&M meetings, annual review meeting added in 2017)

Key: PPIP, Perinatal Problem Identification Programme; PHC, primary health care

Data source: Population, number of PHC clinics and number of staff from District Health Reports 2018/19;[1-3] annual births and perinatal mortality rate from PPIP databased (accessed 4 March 2022); year perinatal audit started from key informant interviews

Figure S1.1 Map of PPIP regions in the Western Cape, South Africa

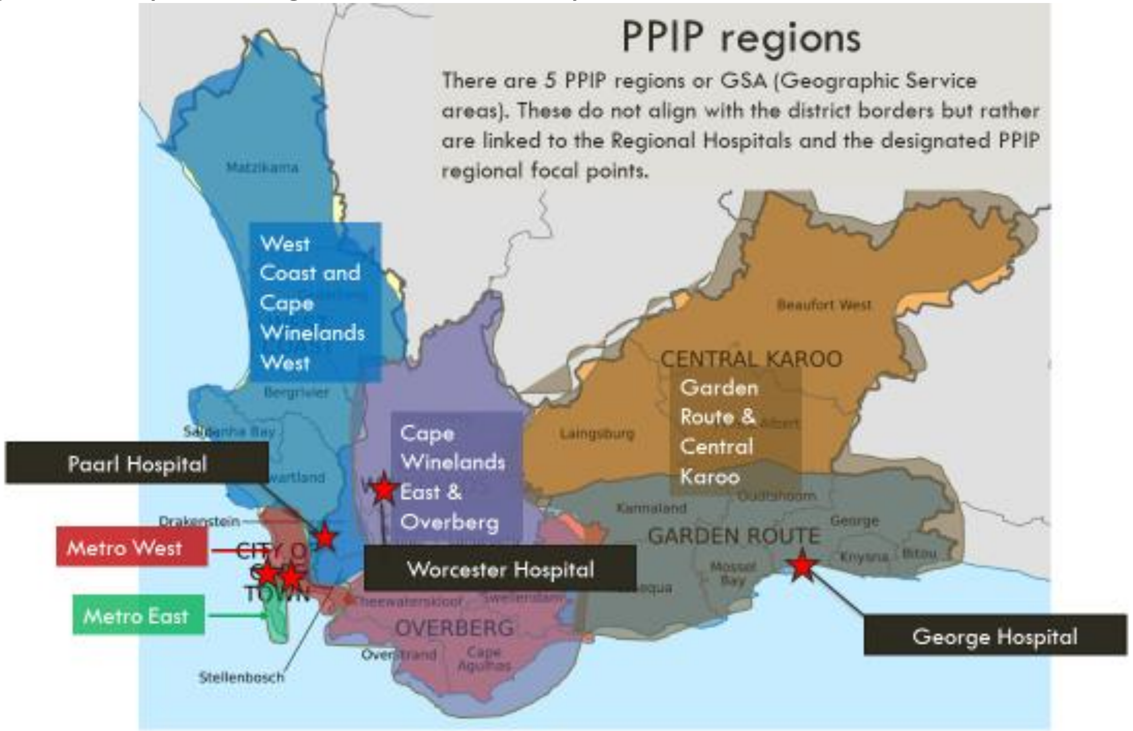

★ Red star signifies referral hospital

Supplementary file 2: Distribution of participants and meeting observations

Table S2.1: Distribution of participants and meeting observations

		District/Regional	Case A	Case B	Case C	Case D	Total
Meeting observations	TOTAL MEETINGS	2	1	1	3	0	7
	PPIP provincial meeting	2					2
	M&M meetings		1	1	1		3
	M&E meetings				1		1
	Other meetings				1		1
	TOTAL INTERVIEWS	5	10	11	10	5	41
Provincial, regional and district level	District manager	0					
	Other district staff	1					1
	Provincial actors	2					2
	Regional PPIP focal persons	2					2
Sub-district level	Clinical manger		1	0	1	0	2
	Comprehensive service manager		0	0	0	0	0
	Information manager		1	1	0	1	3
	Information officer		0	0	1	1	2
	Medical manager		1	1	n/a	1	3
	Nursing manager		1	1	1	1	4
	Pharmacy supervisor		0	0	0	0	0
	PHC manager		0	0	1	0	1
	Quality assurance manager		n/a	0	1	0	1
Facility level	Enrolled nurse		0	0	0	0	0
	Family physician		1	1	1	0	3
	Medical officer (incl Senior and Registrar)		0	2	2	0	4
	Operational manager (facility)		n/a	1	n/a	n/a	1
	Operational manager (maternity)		1	n/a	1	1	4
	Professional Nurse		2	2	1	0	5
PHC level	PHC clinic manager		1	1	0	0	2
	PHC nurse practitioner		1	1	0	0	1

Note: n/a indicates that this position did not exist at this site or was not occupied at the time of the data collection.

Key: M&E, monitoring and evulation; M&M, morbidity and mortality; PPIP, Perinatal Problem Identification Programme; PHC, primary health care

Supplementary file 3: Data collection tools

A. FACILITY LEVEL KEY INFORMANT SEMI-STRUCTURED INTERVIEW GUIDE

BACKGROUND

1. What is your position, how long have you held it and what in summary are your main responsibilities?

PURPOSE OF PERINATAL DEATH REVIEW

2. Can you please describe the perinatal death review process from your perspective?
    a. *Probes:*
        i. *Audit cycle: Data collection, case preparation, M&M meeting, PPIP entry, reports, feedback meetings?*

3. Can you please explain from your perspective the purpose of the perinatal death review process?
    a. *Probe:*
        i. *Why does it exist and what problems does it aim to solve?*
    b. Follow up questions:
        i. Do you think staff agree on the purpose and benefit of using the process to prevent mortality and morbidity?

YOUR ROLE IN THE PERINATAL DEATH REVIEW PROCESS

4. What are your responsibilities relating to the implementation of the perinatal death review process?
    a. *Probe:*
        i. *What are your specific roles: administration of PPIP, preparation for M&M meetings, attending meetings, etc…*
    b. Follow up questions:
        i. How do these responsibilities relate to the rest of your work? Are the responsibilities aligned with your work?
        ii. *Is it part of your official job description?*

PURPOSE, FUNCTIONING AND RESOURCING OF PERINATAL DEATH REVIEW

5. Can you please describe your thoughts on how the process is actually working in this facility?
    a. *Probes:*
        i. *Are the correct people assigned to implement process?*
    b. Follow up questions
        i. How are the results of the perinatal death review process shared internally at facility, between levels of health system? Feedback at meetings, emails, other?

6. To what extent do you think people learn from the perinatal death review process and then modify their work in response to the feedback?
    a. *Probes:*
        i. *Do you have any recommendations for how to improve feedback to you?*
        ii. *Why do you think it is worth the investment and effort as a team? As an individual?*
        iii. *Can you describe barriers that have prevented successful implementation? What has been done in the past to overcome some of these barriers?*

7. What resources are available to sustain the process of implementing perinatal death reviews in terms of money, strategy and other resources?
    a. *Probes:*
        i. *What funding is available to implement the actual review process? Where is it budgeted? Is this sufficient?*
        ii. *How do you feel supported by hospital management and district management to implement?*
        iii. *What training and supportive supervision do and other staff you receive? Is this sufficient?*
        iv. *Are there additional tools or strategies that have been implemented to achieve successful implementation and sustainability of audit programmes?*

YOUR THOUGHTS ON HOW THE TEAM IMPLEMENTS PERINATAL DEATH REVIEWS

8. Can you describe how the team is organized or works together to implement perinatal death reviews at this facility?
    a. *Probes:*
        i. *How do people on the team support one another? Give example*
        ii. *Do you think people buy into the process of perinatal death reviews - Why?*
        iii. *Are people open to new ways of working together to strengthen implementation of the perinatal death review process? Give example*
        iv. *Are the correct people assigned to the related tasks?*
    b. *Reserve for people with very specific responsibilities, e.g.*
        i. *How does the core PIPP team support each other? [Refer back to responsibilities mentioned and try to link them in probes for 'cross-over' support.]*
        ii. *What kind of support do you get from staff members who are not intensively involved with the PPIP process but who attend meetings? Do you ever need / ask for assistance or information from staff members who are not intensively involved with the PPIP process but who attend meetings? If yes [without elaboration]: ask for examples.*
    c. *For staff not very aware of processes:*
        i. *Does XXX [name of the person in ward / in-charge / nursing manager more intensively involved in PPIP processes] ever ask you for assistance or help with information or performing tasks needed for PPIP? If yes [without elaboration]: Can you describe in more detail? or Can you recall any specific occasions?*

9. Who are the key people who drive the process at this facility and can you describe how they interact with others on the team?
    a. *Probe:*
        i. *How do they manage the process?*
        ii. *How do they get others involved?*
        iii. *What motivates these "drivers" or agents of change to support sustained practice of perinatal death audits?*
        iv. *Ask for examples or specific incidents / occasions the participant can recall where the driver(s) displayed this passion or made specific statements about wanting to ensure health mother/baby outcomes. (Some drivers may have repetitive behaviours, e.g. repetition of phrases that demonstrate these. If I had been a*

driver, they would have recalled phrases like "If it is not recorded, it has not been done" or "Never give up!")

v. *What kind of leadership qualities do you think the PPIP driver(s) have? Look out for examples / events / incidents recalled – could be outside the narrow PPIP focus / more general qualities or events.*

10. I want to better understand the trust you have in yourself and in other's regarding perinatal death review. With that in mind:
    a. Two questions:
        i. How confident are you in your ability to implement your functions relating to the perinatal death review process?
        ii. How confident are you in other team member's ability to implement their responsibilities?
    b. *Probes:*
        i. *Does the process ever threaten trust between staff members? Ask for example*
        ii. *Can you give an example of a time a team member surprised you with their ability to perform or not a responsibility linked to the perinatal death review process?*

YOUR OVERALL REFLECTIONS OF PERINATAL DEATH REVIEWS IN CONTEXT

11. When you reflect about the perinatal death review process, in what ways do you think it evolved or changed over the years?
    a. *Probe:*
        i. *Membership, frequency of meetings, composition of attendees to meetings, upgrades to PPIP, use of DHIS*

12. What would be your recommendations for improving future implementation of perinatal death reviews in your facility?
    a. *Probes:*
        i. *What could be done to further facilitate implementation and sustainability? [Add a few specific more probes around "components" and "actions", e.g. the meetings, the system, the process, the software, people involved, etc]*
        i. *Are there components and/or actions about these audits, e.g. the meetings, the system, the process, the software, people involved, etc, that you would change and why?*

13. In your facility, you have the perinatal death audit, but also other death audits processes (e.g. CHIP), quality improvement processes and accountability mechanisms.
    What are the key processes or mechanisms in place that you think work best for improving the care in your facility and why? Also how does it link, if at all, with perinatal death reviews?
    a. Probe:
        i. *Is there value in doing perinatal death reviews in the context of these other efforts?*
        ii. *Can you describe the linkage between the perinatal death audit process and these* other initiatives? *Are there overlaps? Are they repetitive? Reinforcing? Tell me more*

14. Are there any other factors that you think drive how perinatal death reviews functions in your facility that we have not yet discussed?

LAST QUESTION

15. Are there any other issues around PPIP or death audits that you think would be useful for me to know of?

B. KEY INFORMANT INTERVIEW COVERSHEET AND REFLECTIONS

COVERSHEET

**1. Details of interview**	
Unique individual ID	
Date of interview	
Time of interview	
Place of interview	
Interviewer	

**2. Level**		**3. Respondent type**	
National/Provincial		*Regional PPIP manager*	
District		*District manager*	
Sub-District		*District MWCH manager*	
Hospital		*District Quality assurance manager*	
Other		*CEO hospital*	
**4. Consent to record**		*Clinical staff (doctor)*	
Yes – written		*Clinical staff (nurse/midwife)*	
Yes – oral		*Information manager*	
No		*M&M leader*	
N/A		*Other (specify)*	

DEBRIEF

Step 1: Reminder:

- Upload audio into dropbox folder
- Place the consent forms in a safe location
- Complete the reflection notes
- Fill in the data management spreadsheet

Step 2: Complete descriptive field notes

A. Provide physical description of informant

B. Provide physical description of interview location (e.g. Office, skype, telephone, boardroom)

C. Provide overview of interview logistics and feasibility
   a. How feasible was it to find and interview respondents in a private setting?

   b. Were there any issues with the recording quality and notetaking?

c. Were there any issues with the interview length? If so, what should be considered in the future?

Step 3: Complete reflective field notes

D. Reflective commentary
   a. Overall perceptions of the interviews: did it go well? Instances of excellent probing, active listening, managing time well and keeping the interview on track?

   a. Were any follow ups that the interviewer or respondent agreed to take forward? Did the informant recommend any additional individuals to interview?

   b. Content of data
      - Impressions regarding the intensity of involvement of informant in perinatal death reviews

      - Discuss the key domains, noting *major themes emerging* from today's research and noting whether some of the domains were *skipped or not explored in depth*

E. What was the role or stance of the researcher in relation to the setting and participants?
F. Were there any moments of discomfort or discontinuity? Explain?
G. Were there any ethical dilemmas experienced?
H. Did you experience any methodological challenges and obstacles?

   a. Instances of interviewer fatigue or distraction?

   b. Issues with asking many yes/no questions without giving the respondents opportunities to speak at length? How could we better elicit long, descriptive, insightful responses?

   c. Issues of failing to follow the guide? Going off topic? Skipping around too much rather than moving topic by topic?

I. Did you have any revelations and epiphanies?
J. Other

C. OBSERVATION COVERSHEET AND REFLECTIONS

COVERSHEET

**1. Details of observation**	
Unique observation ID	
Date of observation	
Time of observation (HH:MM to HH:MM)	
Place of observation	
Observer	

**2. Level**	**Name of level**	**3. Observation type**	**Name of observation**
National/Provincial		*Meeting*	
District		*Facility ward*	
Sub-District		*Data collection*	
Hospital		*Other*	
Other			
**4. Consent to observe**			
Yes - written			
Yes - oral			
No			
N/A			

WHAT TO OBSERVE DURING THE OBSERVATION[1]

**Category**	**Includes**	**Researcher should note**
Appearance of participants	Clothing, age, gender, physical appearance	Anything that might indicate membership in groups or in sub-populations of interest to the study, such as profession, social status, socioeconomic class, religion, or ethnicity
Verbal behavior and interactions	Who speaks to whom and for how long; who initiates interaction; languages or dialects spoken; tone of voice	Gender, age, ethnicity, and profession of speakers; dynamics of interaction
Physical behavior and gestures	What people do, who does what, who interacts with whom, who is not interacting	How people use their bodies and voices to communicate different emotions; what individuals' behaviors indicate about their feelings

[1] Taken from Mack et al. 2005. Qualitative Research Methods: A DATA COLLECTOR'S FIELD GUIDE. FCI360. https://www.fhi360.org/sites/default/files/media/documents/Qualitative%20Research%20Methods%20-%20A%20Data%20Collector's%20Field%20Guide.pdf

		toward one another, their social rank, or their profession
Personal space	How close people stand to one another	What individuals' preferences concerning personal space suggest about their relationships
Human traffic	People who enter, leave, and spend time at the observation site	Where people enter and exit; how long they stay; who they are (ethnicity, age, gender); whether they are alone or accompanied; number of people
People who stand out or are silent	Identification of people who receive a lot of attention from others OR people who do not receive any attention from others	The characteristics of these individuals; what differentiates them from others; whether people consult them or they approach other people; whether they seem to be strangers or well known by others present

TEMPLATE TO USE FOR NOTETAKING DURING OBSERVATION

**Category**	**Notes from observation**
List of participants	
Appearance of participants	
Verbal behavior and interactions	
Physical behavior and gestures	
Personal space	
Human traffic	
People who stand out or are silent	

DEBRIEF

Step 1: Reminder:

- Scan or type up handwritten notes and place in dropbox folder
- Place the consent forms, if applicable, in a safe location
- Complete the reflection notes
- Fill in the data management spreadsheet

Step 2: Complete descriptive field notes

A. Provide physical description of the observation location (e.g. Office, skype, telephone, boardroom)

B. Provide overview of observation logistics and feasibility
   d. How feasible was it to observe (eg was there space for you, were you able to find the room/location)?
   e. Were there any issues with the notetaking?
   f. Were there any other issues? If so, what should be considered in the future?

Step 3: Complete reflective field notes

C. Reflective commentary
   a. Overall perceptions of the observation: did it go well?
   b. Were any follow ups from the observation to take forward?
   b. Content of data
      - Impressions regarding the intensity of involvement of participants in the observation item?

      - Discuss the key domains, noting *major themes emerging* from today's research and noting whether some of the domains were *skipped or not explored in depth*

D. What was the role or stance of the researcher in relation to the setting and participants?
E. Were there any moments of discomfort or discontinuity? Explain?
F. Were there any ethical dilemmas experienced?
G. Did you experience any methodological challenges and obstacles?
H. Did you have any revelations and epiphanies?
I. Other

Supplementary file 4: Analysis framework with constructs

Table S4.1: Adapted extended Normalization Process Theory constructs, definitions and questions relating to perinatal audits

Concepts	Dimensions	Explanation	Question seeking to answer in this analysis
Emergent expressions of agency	Capability	The capability of participants to enact the intervention depends on its workability and integration into everyday practice	How do people integrate the work into their daily practice? Or how is it not integrated?
	Contribution	The implementation of an intervention over time depends on participants' contributions to enacting it by investing in meaning, commitment, effort and appraisal	Why do people contribute to implementation of the intervention? Or, why don't people contribute?
Dynamic elements of context	Potential	Translating capacity into action depends on participants' commitment to operationalize the intervention	Why are people committed to operationalizing the intervention? Or, why are people not committed?
	Capacity	Implementing an intervention depends on participants' capacity to co-operate and co-ordinate their actions within a social system	What gives people the capacity to implement the intervention? Or what limits people's capacity?

Table S4.2: Extended Normalization Process Theory constructs, definitions and questions relating to perinatal audits

Concepts	Dimensions	Construct according to NPT	Questions relating to construct	Components of construct
Emergent expressions of agency	Contribution	Coherence	Why do people contribute to the intervention?	Direct value of contribution<br>• purpose of intervention<br>• benefits of doing it
		Cognitive participation & Collective Action	How do people collectively organize to implement the intervention?	Team environment (camaraderie, team building)<br>Relational aspects of implementation (mutual respect, relatable, trust, collective responsibility, professional hierarchies, accountability)
		Reconfiguration	How is the intervention helping people to change the way they function and socially interact?	Indirect value of contribution<br>• Intervention's impact on team dynamics<br>• Intervention's impact on clinical practice or on intervention practice.
	Capability	Workability	How are people assigned, trained and supported to implement perinatal audits?	Part of daily work
		Integration	How do people integrate or link the intervention to other activities?	Job description, orientation, embedded into or linked to other work (other meetings, other programmes)
Dynamic elements of context	Capacity	Material and human resources	What resources are in place to support people in the implementation of the intervention?	Any factors relating to health system overall:<br>Human resources - team expansion, tenure/continuity, competency<br>Material resources - resources available, equipment, drugs, building<br>Population need - population growth, burden -HIV prevalence
		Information use	At a broader level, how do people manage, use and access data?	Data systems, data use (observation from meetings and site visits)
		social norms & roles	What are the social norms and roles broadly and related to the intervention?	Management, communication, organizational culture
		Facilitation	How are the review meetings facilitated?	Facilitation of meetings
	Potential	Individual motivation	What motivates people to implement intervention?	Passion for maternity, passion for high quality care
		Shared commitments	Do people have a common goal when implementing the intervention?	Buy-in, ownership, worth the investment
Context of setting	Context of setting	setting	What is the setting where implementation is occurring?	Population, disease burden, number of clinics
		Human resources	What are the human resources available where the intervention is being implemented?	Number of staff, staff rotation, tenure

Concepts	Dimensions	Construct according to NPT	Questions relating to construct	Components of construct
Implementation process	Implementation process	Intervention process	What is the intervention and how is it operationalized?	Identification, review, report, response (descriptive only)
		People involved	Who is involved in the implementation process?	Staff engaged, identified leaders/champions
		resources needed	What resources are needed to implement?	Budget, costs
		History	What is the history of implementation?	Introduction, change over time

Supplementary file 5: Code of conduct example

Figure S5.1: Code of Conduct PPT slide from one sub-district M&M Meeting

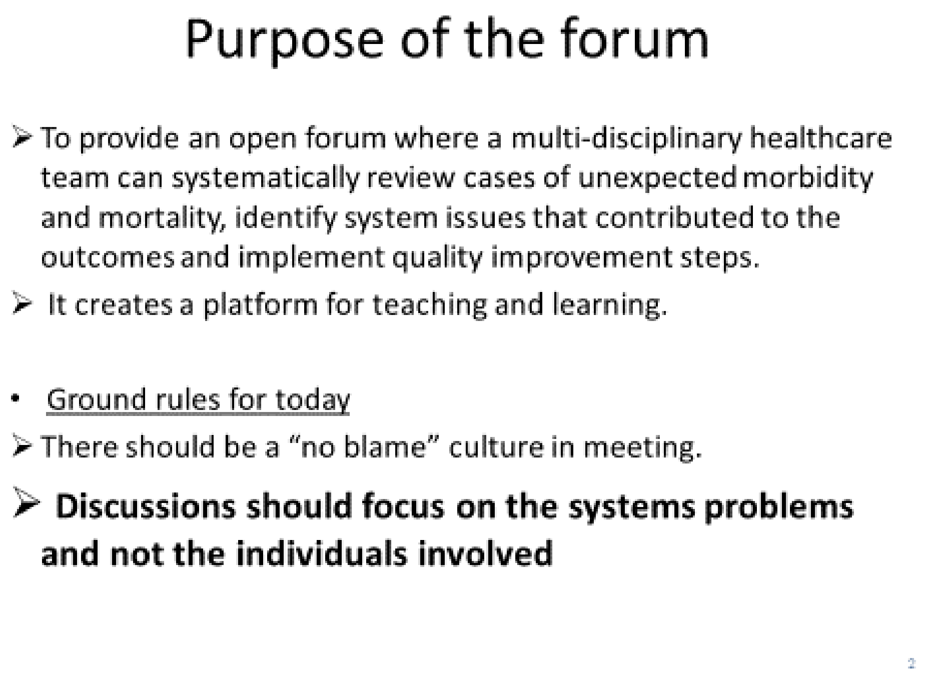

Supplementary file 6: Observed example of a well-facilitated a perinatal death review meeting

Steps of a case review from one case study M&M meeting:

- Step 1: Doctor who had been assigned to maternity that month prepares and reads the case summary; at same time, the facilitator (in this case also the Outreach Specialist) opens the case file and silently reviews the case.
- Step 2: Facilitator identifies key issues relating to the cause of death (if clear). He summarizes what happened in the specific case under discussion according to case file. He sticks to the facts in the case file and does not speculate what could have gone wrong in this specific case nor does he call on those involved to explain what happened.
- Step 3: Based on the main issue identified in his review, he then gives a broader, detailed lesson related to this issue (e.g. case management of a prolapsed cord). As part of his lesson, the facilitator shares a success story from his experience around the same topic to demonstrate that it can be done.
- Step 4: Facilitator then looks at other aspects of the case to see if other gaps can be observed for discussion. In every case, there are other gaps identified with lessons in order to show everyone can always make improvements. Even small things, such as handwriting legibility and quality of information/data entry on standard forms, are flagged.
- Staff are given opportunity to input and ask questions throughout steps 2-4. Actions required by staff also verbalized throughout process but are mostly broad statement e.g. “Sisters, please ensure you fully complete the delivery form even if macerated stillbirth.”
- Step 5: He then indicates orally what is the recommended main cause of death (COD) code so all can hear and agree. If there is not agreement, there is an open discussion until an agreement is reached. His decision is final. He writes the COD code on the PPIP form.
- Step 7: Next case is reviewed in same way.

Supplementary file 7: Mapping of specific factors by case study

Table S7:1 Explanatory factors enabling sustained practice

eNPT dimension and research question	Main results across case studies	Specific factors enabling sustained practice by case study	Case A	Case B	Case C	Case D
**Capability**: implementation depends on its workability and integration into everyday practice.<br><br>*How do people integrate the work into their daily practice? Or how is it not integrated?*	People have the capability to implement because activities related to perinatal audit are integrated and embedded into everyday work.	Activities are part of daily workflow.	✓	✓	✓	✓
		Activities are part of jobs.	✓✓	✓	✓	✓
		Activities are part of orientation.	✓	-	✓	-
		Activities are part of formal training for some.	-	-	✓	✓
		Activities (esp. response) are linked to other meetings and QI processes.	✓✓	✓✓	✓✓	✓
		Activities are integrated with the data system and process (e.g. M&E, information unit).	-	-	✓	✓
		Activities (including response) are part of district support / regional outreach.	✓	✓	✓	✓
		Related costs (people time, responses, meetings) are integrated into existing budgets.	✓	✓	✓	✓
**Contribution**: implementation depends on people's contributions to doing the intervention by investing meaning, commitment, effort and appraisal.	People contribute to the intervention because they understand perinatal audit, value it, trust it and use it to help build and nurture relationships.	People have a common understanding of it.	✓	✓	✓	✓
		People value it for<br>• improving service delivery,<br>• helping them learn skills,<br>• enabling them to debrief as a team.	✓<br>✓<br>✓	✓<br>✓<br>✓	✓<br>✓<br>✓	✓<br>✓<br>-

**eNPT dimension and research question**	**Main results across case studies**	**Specific factors enabling sustained practice by case study**	**Case A**	**Case B**	**Case C**	**Case D**
Why do people contribute to implementation of the intervention? Or, why don't people contribute?		People use the review process as an opportunity to navigate professional hierarchies, hold each other accountable, improve communication, and build/nurture their relationship with team members.	✓✓	✓	✓✓	✓
		People trust the process because the meetings are well facilitated and occur in an environment conducive to learning in a safe, non-blame environment.	✓✓	✓✓	✓	✓
		People also learn overtime that the system works.	✓	✓✓	✓✓	✓✓
**Potential**: implementation depends on people's commitment to operationalizing the intervention.<br><br>*Why are people committed to operationalizing the intervention? Or, why are people not committed?*	People are passionate about their work, committed to improving the quality of service delivery and motivate each other to implement activities relating to perinatal audit.	They are passionate about their work.	✓✓	✓	✓	✓
		They are committed to providing high quality service delivery.	✓	✓	✓	✓
		Their individual motivation stems from the desire to learn, problem solve and self-improve.	✓✓	✓	✓✓	✓
		There are intangible incentives to attend the M&M meetings, i.e. learning, debriefing, communicating.	✓	✓	✓	✓
		There are tangible incentives to attend the M&M meetings, i.e. performance reviews and CPD points.	✓	-	✓	✓
		There is shared commitment to work together and improve the health system because people are invested in the area (e.g. come from community or intend to	✓	✓✓	✓	✓

eNPT dimension and research question	Main results across case studies	Specific factors enabling sustained practice by case study	Case A	Case B	Case C	Case D
		continue working at the hospital for long time).				
		Engagement of multiple actors enables shared commitment to implementation; when some actors are absent from the process, it makes it difficult to implement effectively.	✓ (informal team of 2-3 actors)	✓ (informal team of 2-3 actors)	✓ (informal team of 4-6 actors)	✓ (informal team of 2-3 actors)
**Capacity**: implementation depends on people's capacity to co-operate and co-ordinate their actions.<br><br>*Why are people committed to operationalizing the intervention? Or, why are people not committed?*	People have the capacity to implement because they work in an enabling environment that supports the implementation of perinatal audits.	They work in a well-functioning hospital with sufficient and well managed material and human resources.	✓✓	✓✓	✓✓	✓
		There is a culture of data use for decision-making.	✓	✓	✓✓	✓
		There is a strong, predictable and open communication system in place between levels and staff.	✓✓	✓	✓	-
		There is a strong social network among the staff.		✓✓		
		Good management enables a healthy organizational culture conductive to learning, innovation and accountability.	✓✓	✓	✓✓	✓

Key: eNPT, extended Normalization Process Theory; QI, quality improvement; M&E, monitoring and evaluation; M&M, morbidity and mortality; ; CPD, continuous professional development
